# ENGAGEMENT OF NURSING HOME COMMUNITY EXPERTS TO MODIFY THE DIGNITY INTERVENTION

**DOI:** 10.1093/geroni/igad104.1910

**Published:** 2023-12-21

**Authors:** Liza Behrens, Kalei Kowalchik, Marie Boltz, Jennifer Kraschnewski

**Affiliations:** Pennsylvania State University, State College, Pennsylvania, United States; Pennsylvania State University, University Park, Pennsylvania, United States; Pennsylvania State University, State College, Pennsylvania, United States; Penn State College of Medicine, Hershey, Pennsylvania, United States

## Abstract

Voices of Nursing Home (NH) residents, family caregivers, staff and leaders (community experts) are underrepresented in research development but integral when developing interventions for implementation these complex organizations. This study aimed to explore opinions of NH community experts in the development of a novel person-centered risk management intervention for residents with dementia. Community engagement studios (CES) engaged 62 community experts to gain their perspectives on the DIGNITY (Decision-making in aging and dementia for autonomy) study procedures and instruments. CESs (N=4) were led by an experienced moderator and co-moderator, recorded, and transcribed verbatim. Content analysis was used to summarize responses in a rapid feedback loop. Descriptive statistics of standard measures describe community experts’ ratings of acceptability, appropriateness, and feasibility of DIGNITY intervention. Most community experts identified as female (85%), ranging from 22 - 86 years. Participants were residents with dementia (n=4); family caregivers (n=4); NH administrators (n=16), care manager/ supervisors (n=10), direct-care workers (n=6) licensed healthcare providers (n=6), other NH staff (n=8), ombudsman (n=4) and state surveyors (n=4). Community experts provided suggestions to adapt the study instruments and delivery methods. The study team made changes to all six intervention elements based on expert feedback. Most experts agreed that the DIGNITY intervention was acceptable (M=17.7, SD= 2.08), appropriate (M= 17.24, SD= 2), and feasible (M=16.91, SD= 2.22) to implement in the NH community. Results support practical modifications to the DIGNITY intervention to make it feasible to implement in an upcoming pilot clinical trial.

